# Thyroid-like follicular carcinoma of the kidney: a case report and review of the literature

**DOI:** 10.1186/s13000-014-0186-8

**Published:** 2014-10-08

**Authors:** Merieme Ghaouti, Laurence Roquet, Maximilien Baron, Christian Pfister, Jean-Christophe Sabourin

**Affiliations:** Department of Cytopathology, Charles-Nicole Hospital, Rouen University Hospital, Rouen, France; Department of Urology and Andrology, Charles-Nicole Hospital, Rouen University Hospital, Rouen, France

**Keywords:** Thyroid-like follicular carcinoma of the kidney, Renal cell carcinoma, WHO classification, Metastatic follicular carcinoma of the thyroid

## Abstract

Thyroid-like follicular carcinoma of the kidney is an extremely rare histological variant of renal cell carcinoma. It was described only recently and is not included in the World Health Organization classification of renal tumors. This tumor characteristically shows similar histology to thyroid follicular carcinoma but lacks typical thyroid markers. Herein, we report a new case of thyroid-like follicular carcinoma of the kidney diagnosed in a partial nephrectomy specimen in a 68-year-old-woman. We present typical histological and immunohistochemical findings, discuss differential diagnosis and provide a review of the literature.

**Virtual Slides: **The virtual slide(s) for this article can be found here: http://www.diagnosticpathology.diagnomx.eu/vs/13000_2014_186

## Background

The World Health Organization (WHO) classification of tumors [1] introduced several new distinctive entities of renal cell carcinoma (RCC) in 2004, such as RCC associated with neuroblastoma, Xp11 translocation RCC and mucinous tubular and spindle cell carcinoma. However, since then a potentially new histological entity of renal tumor has emerged: thyroid-like follicular carcinoma of the kidney (TLFCK). This new entity has not yet been integrated into the new WHO classification and is supported by scattered clinical data. TLFCK is an extremely rare tumor with low malignant potential and exhibits morphologic features that strikingly resemble primary follicular carcinoma of the thyroid gland, but TLFCK is characteristically negative for thyroid immunohistochemical markers. The first case of TLFCK was reported in 2004 [2] and since then, to our knowledge, an additional 10 cases have been reported in the literature. We report a further case of this rare histological entity, discuss the clinical, histological and immunohistochemical findings and provide an update on the review of the literature.

## Case presentation

### Case report

**Figure 1:**
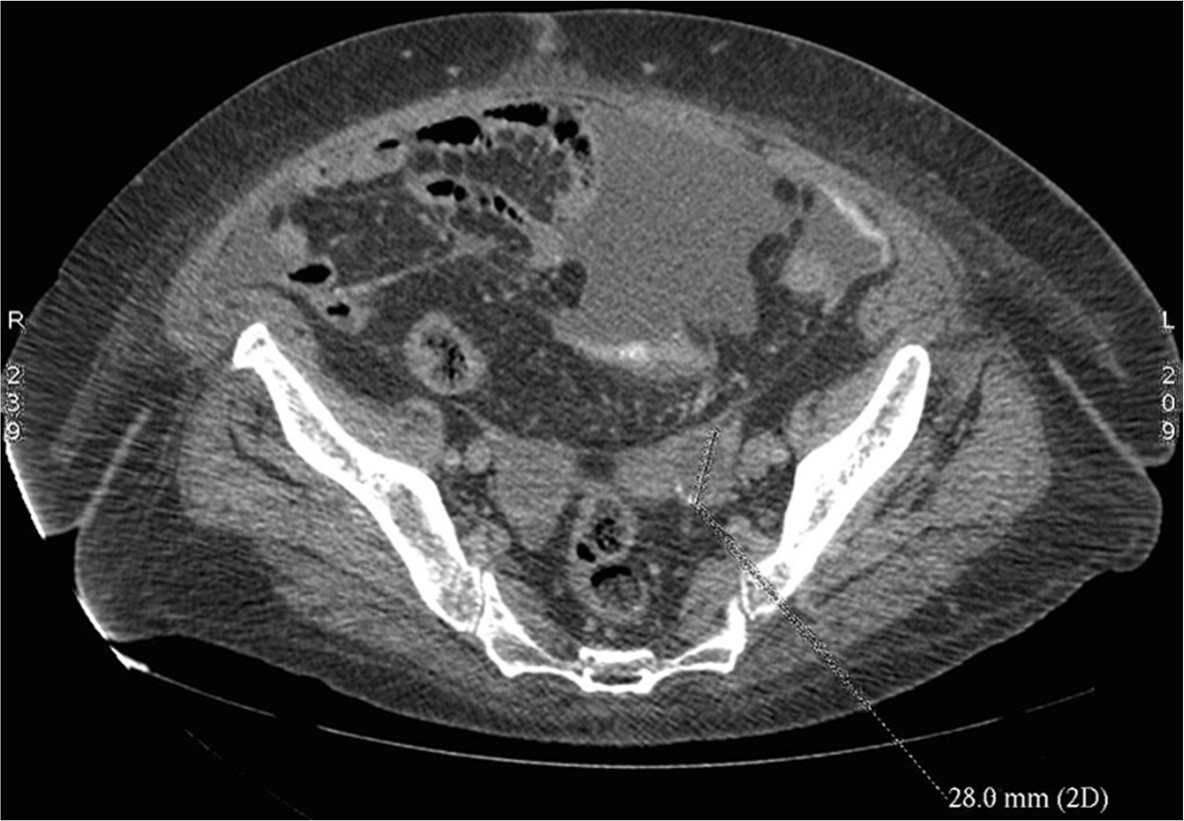
**Axial computed tomography image showing an exophytic lesion of the right kidney.**

A 68-year-old white European presented to her urologist with a long history of relapsing urinary infection, with no hematuria. Except for a uterine prolapse cure 10 years previously, no relevant medical or family history was noted. Physical examination of the thyroid, abdomen and pelvis was normal. Biological data, including thyroid function tests, were within the normal ranges. A computed tomography scan showed a partially cystic enhancing lesion, measuring 11 mm in diameter, arising from the right kidney (Figure 1). Subsequent investigation with a magnetic resonance imaging scan confirmed an 11 mm mass in the mid pole of the right kidney, with no enhancement following gadolinium injection. There was no evidence of either renal vein or lymphadenopathy involvement, or metastatic disease. Given the volume and position of the tumoral lesion, a partial nephrectomy was performed.

**Figure 2:**
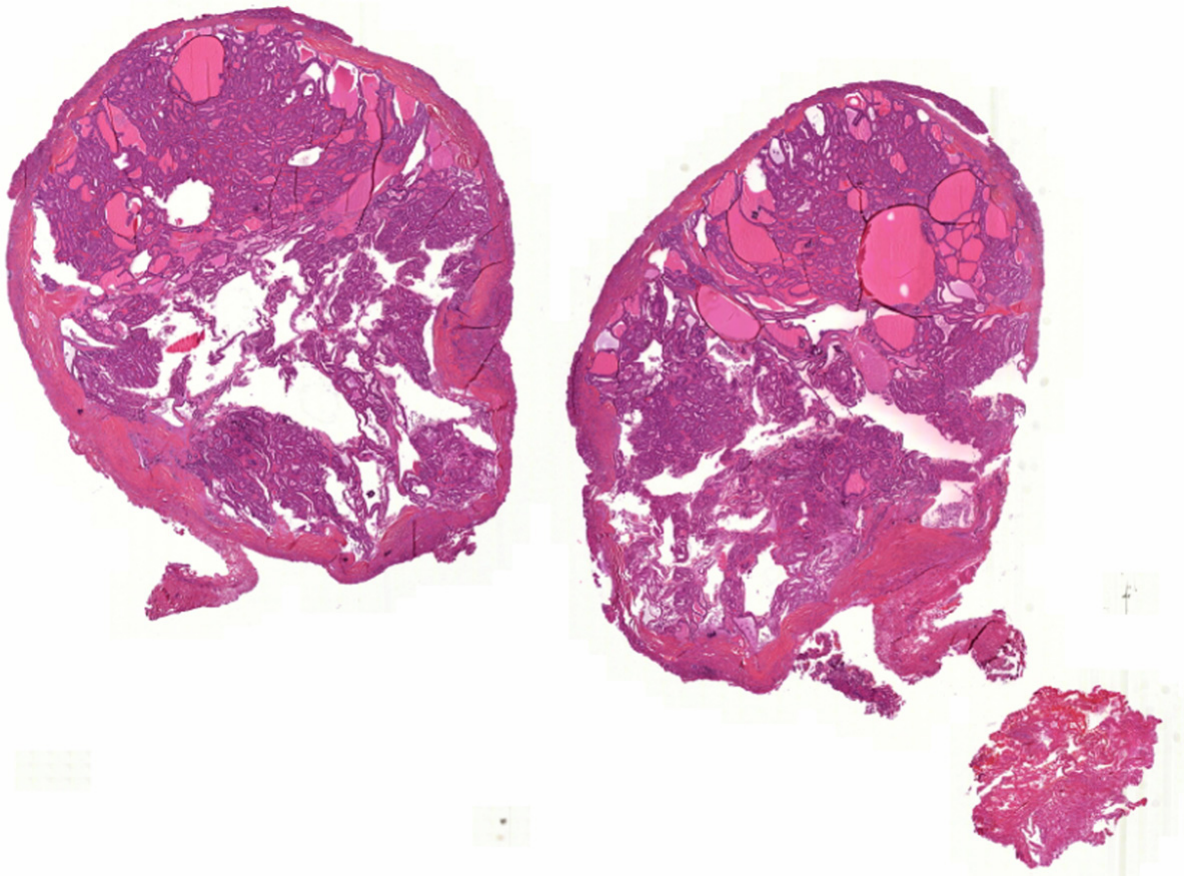
**Representative micrograph of the renal tumor.** The tumor is limited by a thin fibrous capsule (Hematoxylin-eosin, original magnification x10).

**Figure 3:**
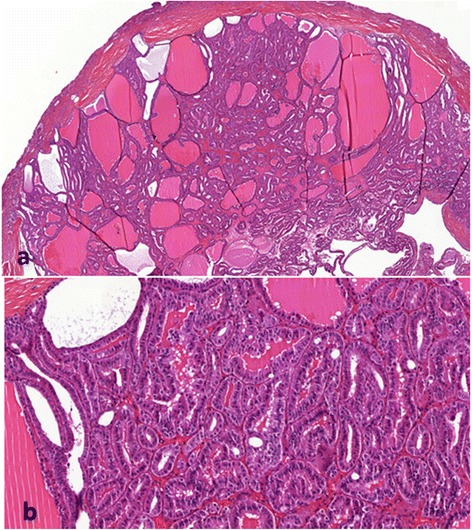
**Representative micrograph of the renal tumor. a**-The tumor is composed of micro- and macro-follicles of varying sizes filled with colloid-like material (Hematoxylin-eosin, original magnification x20). **b**- The follicles are lined by columnar cells with eosinophilic cytoplasm. The nuclei are round with evenly distributed chromatin and inconspicuous nucleoli (Hematoxylin-eosin, original magnification x100).

**Figure 4:**
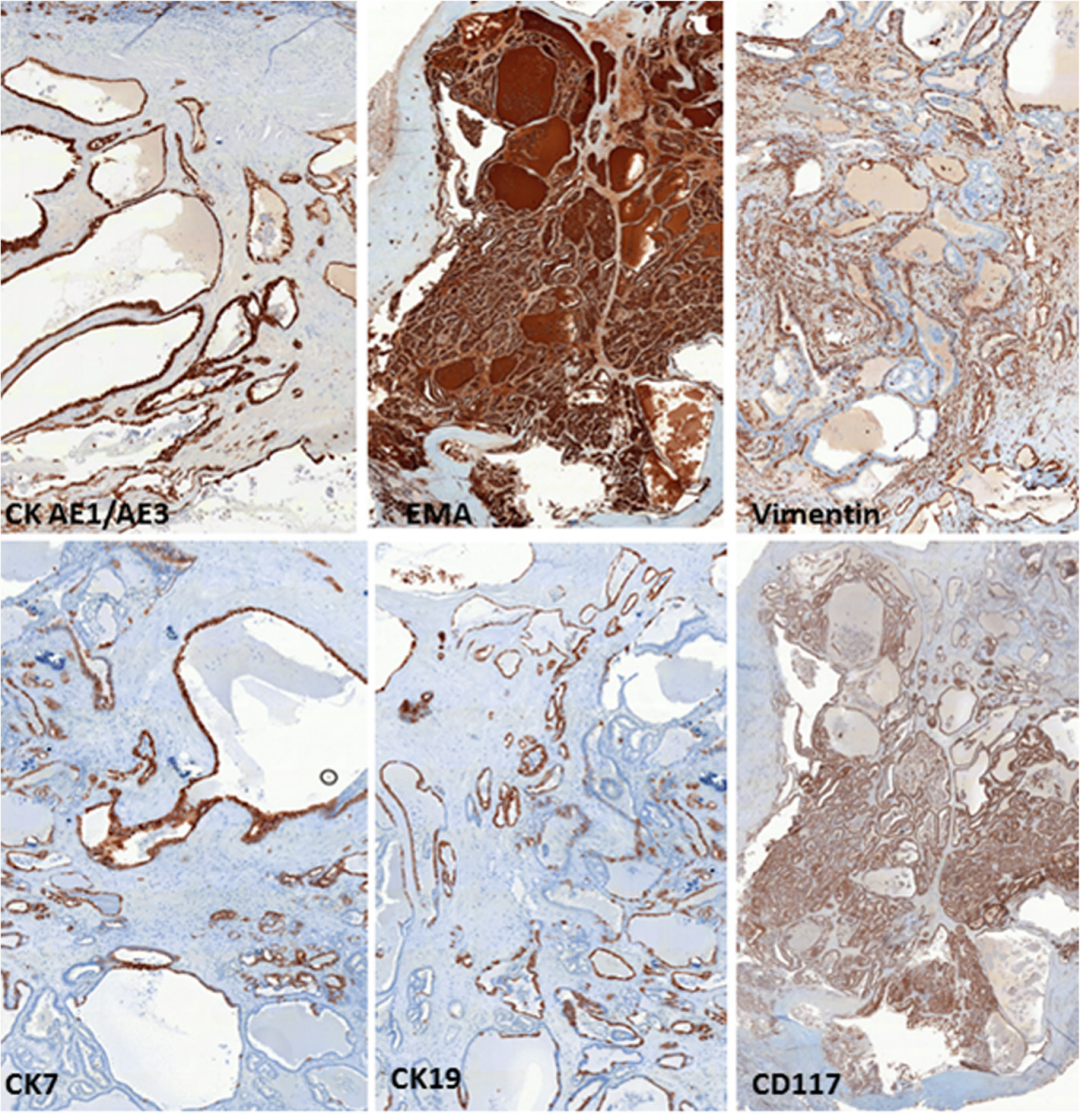
**Immunohistochemical stains.** The tumor cells are positive for CKAE1/AE3, EMA, vimentin, CK7, CK19 and CD117.

Gross examination of the partial nephrectomy specimen showed an 11 mm pale-yellow lesion. The specimen was fixed in 10% buffered formalin, embedded in paraffin and serially sectioned into 4-μm-thick slices. Routine staining with hematoxylin and eosin was performed. Microscopically, the tumor was limited by a thin fibrous capsule (Figure 2). It consisted of macro- and micro-follicles of varying sizes filled with amorphous eosinophilic colloid-like material. Some follicles were cystically dilated (Figure 3a). The follicles were lined by cuboidal to columnar cells with moderate amount of clear or eosinophilic cytoplasm. The nuclei were round to ovoid with evenly distributed chromatin and inconspicuous nucleoli (Fuhrman grade 1–2) (Figure 3b). Mitoses were absent. Focal calcifications were observed within the fibrous capsule of the tumor. No areas of typical conventional (clear cells) or other known types of RCCs were found. Extensive immunohistochemical staining was performed (Table 1). The tumor cells were positive for epithelial membrane antigen (EMA), cytokeratins (CK7, CK19, CKAE1/AE3 and CK34βE12), vimentin and CD117 (Figure 4). The tumor cells were negative for CD10 and 504 s protein. Importantly, staining for thyroid transcription factor (TTF-1) and thyroglobulin (Tg) were negative confirming that the tumor was not a metastatic follicular thyroid carcinoma (Table 1).

**Table 1:** **Antibodies used for immunohistochemical staining and results**

**Monoclonal antibody**	**Source**	**Dilution**	**Result**
EMA	DAKO	1:100	Positive
CK7	DAKO	1:100	Focal positive
CK19	DAKO	1:100	Positive
CKAE1/AE3	DAKO	1:100	Focal positive
CK34βE 12	DAKO	1:50	Focal positive
Vimentin	DAKO	1:100	Positive
CD10	Novocastra	1:50	Negative
CD117	DAKO	1:50	Positive
504 s protein	DAKO	1:200	Negative
TTF-1	DAKO	1:100	Negative
Tg	DAKO	1:400	Negative

Hence, based on the morphology and immunohistochemical profile, a diagnosis of TLFCK was rendered. The final TNM stage with combined imaging was pT1aN0M0.

## Discussion

Primary TLFCK is a rare and recently described entity. Few cases have emerged since the first report by Amin et al. in 2004 [2], with only 11 cases reported in the literature to date. The characteristics of current cases of TLFCK are reported in Table 2.

**Table 2:** **Characteristics of the currently reported cases of TLFCK**

**Cases**	**Age (years)/sex**	**Presentation**	**Tumor size (cm)**	**TNM stage**	**Disease-free at follow-up (months)**
Amin et al. [2]	53/F	Incidental	11.8	pT2Nx	6
Soo et al. [3]	32/F	Incidental	2.1	pT1aNx	54
Amin et al. case 2 [4]	29/F	Incidental	1.9	pT1aNx	84
Amin et al. case 3 [4]	45/M	Incidental	3.5	pT1aN1	17
Amin et al. case 4 [4]	83/M	Incidental	2.1	pT1aNx	48
Amin et al. case 5 [4]	35/M	Incidental	3.0	pT1aNx	20
Amin et al. case 6 [4]	50/M	Incidental	4.0	pT1aN0	7
Dhillon et al. [5]	34/F		6.2	pT1bN2M1	3
Khoja et al. [6]	31/F		4	pT1aN0	21
Malde et al. [7]	29/F		6.5	pT1bN0M0	4
Volavsek et al. [8]	34/M		5.5	pT1bNx	6
Present case	68/F	Incidental	1.1	pT1aN0M0	6

Mean patient age was 34 years (range 29 to 83 years) with no gender predilection (male-to-female ratio 5:7). The tumor was an incidental finding in seven patients, as in our case, but two patients presented with hematuria and two others with flank pain. Mean tumor size was 4.3 cm (range 1.1 to 11.8 cm). With only 11 mm in diameter, our patient’s tumor is the smallest case described, as the diagnosis was performed in a partial nephrectomy specimen. In one case, TLFCK was diagnosed in a patient with polycystic kidney disease [8]. Although the majority of cases were low grade with an indolent course, one patient developed metastatic extension to the renal hilar lymph nodes and another presented with marked symptoms and widespread metastases to the lungs and retroperitoneal lymph nodes. These latter cases provide evidence that this rare variant of RCC has a low but distinct malignant potential and can be clinically aggressive.

On gross examination, all tumors were circumscribed by a fibrous capsule, with or without areas of hemorrhage or necrosis. One tumor protruded into the pelvic cavity [3] and another focally extended into the perinephric tissue. Microscopically, tumors are composed of variably sized follicular structures lined by a single layer of cuboidal to columnar cells with moderate amphophilic to slightly eosinophilic cytoplasm creating macro- and micro-follicles containing inspissated colloid-like material. Marked lymphocytic infiltration may be present in cases of TLFCK, mostly as prominent intratumoral collections. These collections may occasionally contain lymphoid follicles with reactive germinal centers [9]. Our case was devoid of inflammation. The nuclei were round to oval, with evenly distributed chromatin. Most tumors are Fuhrman nuclear grade 2 with occasional grade 3. Mitotic activity is absent or rare. TLFCK immunohistochemical staining results are variably reported: CD10 and PAX2 were positive in 2 out of 10 cases and 1 out of 6 cases, respectively. EMA, CK7 and vimentin showed frequent positivity. PAX8 has been reported positive in tumor cells [5]. Immunostaining for CK19, CK20, and 504 s protein shows variable results. Diffuse positivity for CD117 was detected in our tumor whereas in the literature this marker was always negative when tested. The most important factor in the reported cases of TLFCK is consistent immuno-negativity for the thyroid-specific markers, such as TTF-1 and Tg.

TLFCK should be distinguished from kidney thyroidization and renal metastasis. Thyroidization of the kidney is a well-known phenomenon characterized by atrophic distal tubules or collective ducts with colloid-like hyaline casts imitating the usual structure of the thyroid gland. Usually, thyroidization occurs as a process which is secondary to chronic pyelonephritis or obstructive uropathy and is a common characteristic of end-stage renal disease [10]. However, this is a benign phenomenon that is typically bilateral and widespread, as opposed to TLFCK which presents as a well circumscribed mass and occurs in patients without renal disease, as in our case [7]. Despite its similar appearance, the colloid-like material in TLFCK is composed of Tamm-Horsfall glycoprotein, the most abundant protein in normal urine [11], which is different from thyroglobulin that comprises most of the material in the thyroid follicles [11]. Furthermore, a thyroid-like appearance may occasionally be observed in more well-known other subtypes of RCC, such as clear cell carcinoma, oncocytoma, tubules of papillary RCC, and metanephric adenoma. However, this appearance is rare in these tumors, and if present, it is usually focal. In contrast, TLFCK is composed almost entirely of follicular structures with dense, colloid-like material, with none of the other histological features of these more common RCC subtypes. It is also necessary to exclude renal metastasis, particularly metastatic follicular carcinoma of the thyroid, before performing TLFCK diagnosis. The renal tumor herein described was histologically similar to follicular carcinoma of the thyroid and raised the possibility of metastatic thyroid carcinoma. Follicular carcinoma of the thyroid very rarely metastasizes to the kidney, metastases being usually localized in the lung and bone [3]. Only sixteen cases of follicular thyroid carcinoma metastasing in the kidney have been reported in the literature. In almost all these cases, a primary tumor was present in the thyroid gland, and metastases were widespread, involving other organs [12,13]. Furthermore, all these tumors demonstrated positive immunoreactivity for the thyroid-specific markers TTF-1 and Tg. In contrast, negative immunoreactivity to these markers, combined with reactivity to CK7, CD10 and vimentin, in conjunction with negative clinical and biological thyroid investigations and absence of metastatic disease, allowed us to exclud the possibility of metastatic thyroid follicular carcinoma in our case. Struma ovarii, an ovarian teratoma composed mainly of thyroid tissue might also be considered for differential diagnosis. This ovarian neoplasm is rarely malignant and metastasizes in only 5% of cases, commonly to the peritoneum or liver [14]. To our knowledge, there are no reports of struma ovarii metastasizing to the kidney. In any case, metastatic carcinoma cells from struma ovarii should disclosed positive immunoreactivity for TTF-1 and Tg [7]. In all reported cases of TLFCK, including ours, the carcinoma cells lacked immunoreactivity for these two markers, and no lesion was found in the ovary. The unique histopathological features of our case, combined immunophenotype of the tumor cells, are consistent with previous reports of a rare primary thyroid-like follicular renal cell carcinoma.

In TLFCK, genetic analysis has shown variable genetic alterations [3,4]. Using comparative genomic hybridization analysis, Jung et al. [3] reported losses of chromosomes 1p36, 3 and 9q21-33 and gains of chromosomes 7q36, 8q24, 12, 16, 17p11-q11, 17q24, 19q, 20q13, 21q22.3, and Xp in their case. Using fluorescent in situ hybridization analysis, Sterlacci et al. [15] found losses of chromosomes 1, 3, 7, 9p21, 12, 17, and X in this tumor.

## Conclusion

In summary, we report the case of an unusual renal tumor disclosing histologic features similar to follicular carcinoma of the thyroid, but lacking typical thyroid markers, and corresponding to TLFCK. This recently described histological variant of RCC, not included in the current WHO classification of renal tumors, is important to recognize in order to prevent unnecessary or inappropriate treatment due to misdiagnosis.

## Consent

Written informed consent was obtained from the patient for publication of this case report and accompanying images. A copy of the written consent is available for review by the Editor-in Chief of this journal.

## Abbreviations

WHO: World Health Organization
RCC: Renal cell carcinoma
TLFCK: Thyroid-like follicular carcinoma of the kidney
EMA: Epithelial membrane antigen
TTF-1: Thyroid transcription factor
Tg: Thyroglobulin
